# Combination Treatment of TRPV4 Agonist with Cisplatin Promotes Vessel Normalization in an Animal Model of Oral Squamous Cell Carcinoma

**DOI:** 10.3390/medicina58091229

**Published:** 2022-09-06

**Authors:** Farhana Yahya, Marina Mohd Bakri, Mohammad Zakir Hossain, Syarifah Nur Syed Abdul Rahman, Aied Mohammed Alabsi, Anand Ramanathan

**Affiliations:** 1 Department of Oral and Craniofacial Sciences, Faculty of Dentistry, Universiti Malaya, Kuala Lumpur 50603, Malaysia; farhana.yahya@siswa.um.edu.my (F.Y.); synur@um.edu.my (S.N.S.A.R.); 2 Department of Oral Physiology, School of Dentistry, Matsumoto Dental University, 1780 Gobara Hirooka, Shiojiri, Nagano 399-0781, Japan; mohammad.zakir.hossain@mdu.ac.jp; 3 Department of Oral Biology and Biomedical Sciences, Faculty of Dentistry, MAHSA University, Jenjarom 42610, Malaysia; aied@mahsa.edu.my; 4 Department of Oral and Maxillofacial Clinical Sciences, Universiti Malaya, Kuala Lumpur 50603, Malaysia; drranand@um.edu.my; 5 Oral Cancer Research and Coordinating Center, Universiti Malaya, Kuala Lumpur 50603, Malaysia

**Keywords:** oral squamous cell carcinoma (OSCC), transient receptor potential vanilloid 4 (TRPV4), 4-nitroquinoline 1-oxide (4NQO), angiogenesis, vascular normalization

## Abstract

*Background and Objectives*: Oral squamous cell carcinoma (OSCC) is the sixth most common malignancy in the world. Transient receptor potential vanilloid 4 (TRPV4) channel has been shown to be involved in angiogenesis in multiple types of tumors. However, not much is known about TRPV4′s involvement in OSCC. Thus, in this study, we investigate the effect of administering a TRPV4 agonist on angiogenesis in OSCC. *Materials and Methods:* Thirty-six Sprague Dawley (SD) rats were used in this study. 4-nitroquinoline 1-oxide (4NQO) was used to induce OSCC. Cisplatin (an anticancer drug), and GSK1016790A (an agonist for TRPV4) was used in this study. Immunohistochemistry was employed to examine the TRPV4 expression. An RT^2^ Profiler PCR Array was performed for gene expression analysis of TRPV4, vascular growth factors that correspond directly with angiogenesis, such as angiopoietin (Ang-1 and Ang-2), and tyrosine kinase (Tie-1 and Tie-2) receptors. Tumor vessel maturity was assessed by microvessel density and microvessel-pericyte-coverage index. *Results:* RT^2^ profiler PCR array showed significant elevated levels of Ang-1 (2.1-fold change; *p* < 0.05) and Tie-2 (4.5-fold change; *p* < 0.05) in OSCC following the administration of a combination of GSK1016790A and cisplatin. Additionally, the combination treatment significantly reduced the microvessel density (*p* < 0.01) and significantly increased the percentage of microvessels covered with pericytes (*p* < 0.01) in OSCC. Furthermore, tumor size was significantly reduced (*p* < 0.05) in rats that received cisplatin alone. The combination treatment also greatly reduced the tumor size; however, the data were not statistically significant. *Conclusions:* The findings suggest that combining a TRPV4 agonist with cisplatin for treatment of OSCC promote vessels normalization via modulation of Ang-1/Tie-2 pathway.

## 1. Introduction

Oral squamous cell carcinoma (OSCC) is the sixth most common cancer worldwide [1,2]. One of the hallmarks of cancer is angiogenesis, in which the vessels are leaky, tortuous, irregularly shaped, and have scarcer, loosely associated pericytes. Various studies have shown that impaired pericyte coverage on endothelial cells affects the functionality of the vessels, which causes instability of the vessels with a high risk of hemorrhage and metastatic ability [3,4]. The normalization of these vessels involves restoring deformities, both structurally and functionally, and this may lead to an improvement of the tumor microenvironment [5]. The normalized tumor vessels have a well-structured basement membrane with greater pericyte coverage, and better features, such as reduced leakiness, dilation, and tortuosity [6,7].

The transient receptor potential vanilloid (TRPV) family consists of six members, TRPV1-TRPV6. Among these, TRPV4 has recently drawn significant attention for its potential role in calcium signaling and cancer research. The involvement of TRPV4 has been reported for various types of cancer, such as breast, lung, colon, uterus, and stomach [8,9,10,11,12]. However, the involvement of TRPV4 in oral cancer, particularly OSCC, is not fully understood. Given that different types of cancer behave and respond differently to various types of treatment, understanding the effect of TRPV4 activation in OSCC by using a TRPV4 agonist or combining the use of a TRPV4 agonist with an anticancer drug, such as cisplatin, will be of great importance, especially in these times when the options for cancer treatment have diversified. For example, in the case of molecular targeted therapy, this type of treatment has been explored and applied in the clinical setting for treating cancer. Thus, this study aims to explore the involvement of TRPV4 in regulating angiogenesis and the effect of a combination treatment of a potent TRPV4 agonist (GSK1016790A) and cisplatin (a well-known anticancer drug) in an animal model of OSCC.

## 2. Materials and Methods

### 2.1. Animal Care

Experimental procedures were approved by the Institutional Animal Care and Use Committee (IACUC), Faculty of Medicine, University of Malaya, Malaysia (Ethical Approval No-2018-190502/DENT/R/MMB (M), date of approval: 15 May 2018). All animals were treated humanely according to the guidelines developed by the National Centre for the Replacement, Refinement and Reduction of Animals in Research, ARRIVE (Animal Research: Reporting of In Vivo Experiments).

### 2.2. Administration of 4NQO

The rats received either reverse osmosis water (control) or 4-nitroquinoline 1-oxide (4NQO) supplemented drinking water for 14 weeks; following this, the rats were then reverted back to reverse osmosis water and monitored until week 22 [13]. 4NQO-supplemented drinking water was freshly prepared to a final concentration of 0.02 g/L (20 ppm) and dispensed to the rats in foil-wrapped bottles to hinder possible photodegradation of the carcinogen. Rats were weighed once weekly and sacrificed at 22 weeks.

### 2.3. Experimental Design for Animal Grouping

**Figure 1:**
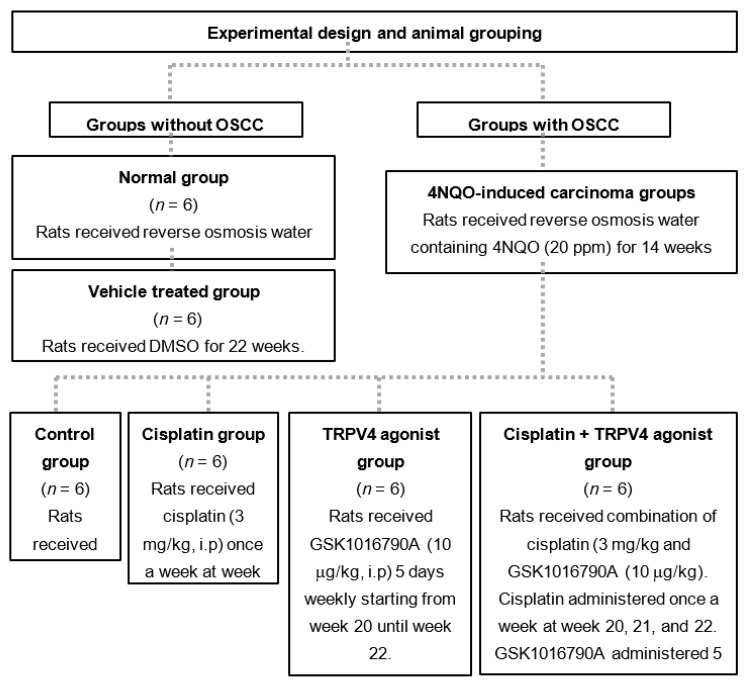
Experimental design for animal grouping involving a total of 36 male Sprague Dawley rats. (Oral squamous cell carcinoma (OSCC), transient receptor potential vanilloid 4 (TRPV4) channel, 4-nitroquinoline 1-oxide (4NQO), Dimethylsulfoxide (DMSO), GSK1016790A (TRPV4 agonist)).

A total of 36 male Sprague Dawley rats were used. The experimental design for the animal grouping is shown in Figure 1. The sample size was calculated using the formula as explained by Ariffin and Zahiruddin (2017) [14], involving the resource equation approach. Based on the formula, the minimal and maximum sample size required for this study were *n* = 4 and *n* = 6 for each group, respectively.

### 2.4. Tumor Assessment and Histology

Tumor volume was measured according to the following formula [15,16]:

π/6 × width × length × height

Half of the tongue was fixed in 10% neutral-buffered formalin and processed for routine hematoxylin and eosin (H&E) staining. A qualified oral pathologist evaluated all the histological slides without prior knowledge of the animal groups.

### 2.5. Administration of Cisplatin and GSK1016790A

Cisplatin was diluted using saline solution, 0.9%, making up the final concentration of 3 mg/kg, and administered to the rats intraperitoneally once a week for three weeks [17]. A stock solution of GSK1016790A (10 mM) was prepared in 1% dimethyl sulfoxide and 10 µg/kg of GSK1016790A14 was administered consecutively for five days a week for three weeks (week 20, 21 and 22) via intraperitoneal injection to the right and left side alternately.

### 2.6. RT^2^ Profiler Arrays

Total RNA was extracted from the tongue using RNeasy Mini Kit (Qiagen, Hilden, Germany). Reverse transcription of RNA to complementary DNA (cDNA) was carried out using RT^2^ First Strand Kit (Qiagen, Hilden, Germany). The real-time PCR was performed using RT^2^ Profiler Arrays together with RT^2^ SYBR Green Mastermixes (Qiagen, Hilden, Germany). Each well in the array plate was prehybridized with a primer pair of gene members, as listed in Table 1. Real-time PCR was carried out using ABI 7500 HT FAST thermal cycler (Applied Biosystems, Waltham, MA, USA).

### 2.7. Single and Double Immunostaining

The primary antibodies used for immunohistochemistry are shown in Table 2. The secondary antibody, MACH 2 Double Stain 2 (Biocare Medical, Pacheco, CA, USA), was applied on the slides and incubated for 30 min. Double staining immunohistochemistry requires the use of two different chromogens, such as 3,3’ Diaminobenzidine (DAB) and warp red (WR) (Biocare Medical, Pacheco, CA, USA). Verification for TRPV4, α-SMA, and CD31 was carried out for every group using a light microscope, and the sections were then digitized using a digital slide scanner, Panoramic Desk Scanner (3DHistech, Budapest, Hungary).

### 2.8. Immunoassay

The tongue tissues samples were homogenized in lysis buffer, RIPA (Sigma-Aldrich, St. Louis, MO, USA) prior to immunoassay procedure. All reagents were prepared according to the manufacturer’s protocol, Rat Vascular Injury Magnetic Bead Panel 1 (Cat. # RV1MAG-26K), Merck, Germany. The plate was run on Luminex^®^ 200™ with the xPONENT^®^ software. The Median Fluorescent Intensity (MFI) data was then analyzed using a 5-parameter logistic.

### 2.9. Digital Histology Scoring

Staining for anti-TRPV4 were analyzed using image software analysis (Fiji, ImageJ, National Institute of Mental Health, Bethesda, MD, USA) [18]. Scoring of DAB intensity was calculated according to the following formula:

f = 255 − i,

f = final DAB intensity, i = mean of DAB intensity obtained from the software. i value ranges from the highest expression of 0 (deep brown) to no expression of 255 (total white).

### 2.10. Microvessel Density and Microvessel-Pericyte-Coverage Index

Microvessel density evaluation was carried out by counting the microvessels in five highest vascular spots within the tumor stained with CD31 marker. Prior to this, the OSCC area was first scanned under low magnification (40× magnification). Photographs of five selected spots were taken at 400× magnification, and an average was calculated. Microvessel pericyte index was performed on double immunohistostained slides. The pericytes were identified as a single layer of α-sma-positive cells in close proximity to the CD31-positive microvessels. The microvessel pericyte index was assessed by counting the microvessels associated with α-sma-positive pericytes over the total number of CD31- positive microvessels. The average percentage of pericyte coverage for five spots were then calculated [19].

### 2.11. Statistical Analysis

The data obtained by RT^2^ Profiler PCR Array were analyzed using Data Analysis software (Qiagen, Hilden, Germany) (http://lc.chat/rt2-pcr-arrays-data-analysis-center) (accessed on 19 March 2019). The *p* values were examined based on a Student’s *t*-test of the replicate 2^−∆Ct^ values for each gene in the control group. The digital histological scoring data for TRPV4, immunoassay data for vascular endothelial growth factor (VEGF), microvessel density and microvessel pericyte index, and tumor volume data were examined using the ANOVA test. The assumptions of univariate normality of residuals and homoscedasticity of residuals were assessed using the Brown–Forsythe test and D’Agostino–Pearson omnibus (K2), respectively. The Post Hoc Turkey’s test was performed for those results that exhibited significant differences in ANOVA. All tests were performed using the GraphPad Prism 8 software (GraphPad Software, Inc., San Diego, CA, USA). The measured values were expressed as a mean ± confidence interval (95% CI). For all statistical analysis, *p* < 0.05 was considered to be statistically significant.

## 3. Results

### 3.1. Histological Features of OSCC Lesions in Rats Induced by 4NQO

**Figure 2:**
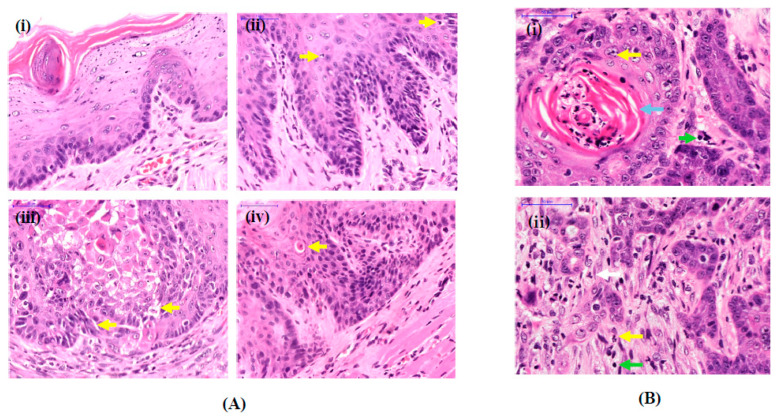
(**A**) Histology of tongue tissue showing (**i**) normal oral epithelia and (**ii**–**iv**) epithelial dysplasia. Features of epithelial dysplasia (shown by the yellow arrows) of animal groups treated with 4NQO; (**ii**) budding of rete ridges and increase in mitotic cells; (**iii**) loss of epithelial cell cohesion and abnormal epithelial stratification; (**iv**) presence of apoptotic cells with eosinophilic cytoplasm and pyknotic nucleus. (**B**) Histology of tongue tissue showing development of OSCC in animal groups treated with 4NQO. Photomicrographs of H&E slides showing (**i**) well-differentiated OSCC with nest and keratin pearl formation (blue arrow); (**ii**) pleomorphic tumor cells showing atypical mitosis (white arrow), multiple nucleoli (yellow arrow), higher nuclear/cytoplasmic ratio and hyperchromasia (green arrow). Scale bar is 50 µm.

Tongue tissues from the untreated group (normal) showed normal characteristics of oral epithelium (Figure 2A(i)). In contrast, tongue tissues from 4NQO-supplemented animals showed features of dysplasia (Figure 2A(ii–iv)) and OSCC (Figure 2B). Tumor cells were pleomorphic with hyperchromatic nuclei, and there was discontinuation at the basement membrane with tumor cells invading the submucosa and the muscles beneath it, creating small nests with formation of keratin pearls (Figure 2B).

### 3.2. TRPV4 Expression in OSCC Lesions

**Figure 3:**
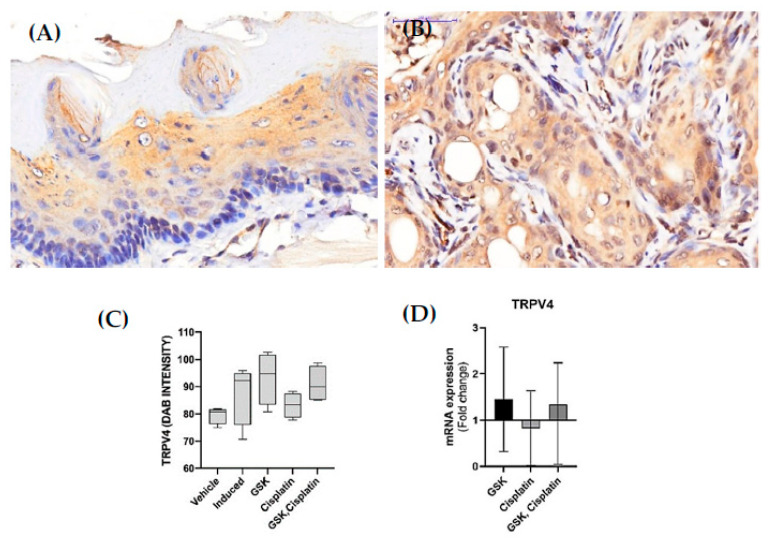
Photomicrographs of TRPV4 expression of (**A**) normal tissue and (**B**) OSCC tissue. (**C**) DAB intensity of TRPV4 expression (**D**) TRPV4 mRNA expression (fold changes relative to values obtained from the OSCC group induced with 4NQO only (set as 1) and normalized by changes in the average value of housekeeping genes (GAPDH, 18 s, and β-actin). Fold-change values greater than 1 indicate an upregulation and vice versa. Scale bar is 50 µm. The data are presented as mean ± CI.

**Figure 4:**
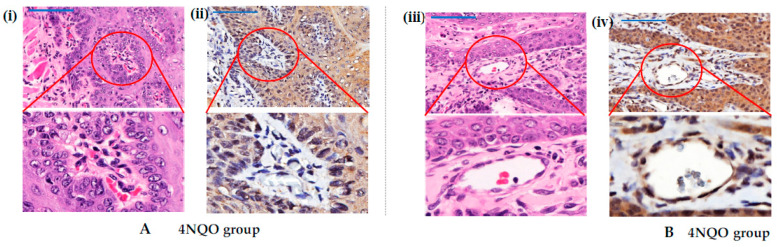
(**A**) TRPV4 expression in blood vessels of tongue tissues with OSCC of animal groups treated with 4NQO. (**i**) H&E and (**ii**) immunohistochemistry staining of blood vessels. (**B**) (**iii**) H&E and (**iv**) immunohistochemistry staining of blood vessels in 4NQO group treated with GSK1016790A. Note that GSK1016790A enhanced the TRPV4 expression in blood vessel of cancerous tissue. Scale bar is 50 µm and magnified to 1:1.

The immunohistochemistry results revealed that TRPV4 was expressed in the membrane and cytoplasm of the squamous cells. (Figure 3A,B).Immunohistochemistry (Figure 3C) and qPCR (Figure 3D) results revealed that TRPV4 mRNA and protein expression in OSCC tissue were upregulated in both groups treated with GSK1016790A (93.27 ± 9.601 (95% CI [77.99, 108.50]), 1.45-fold change) and cisplatin + GSK1016790A (90.93 ± 6.574 (95% CI [80.47, 101.40]), 1.34-fold change), although the data were not statistically significant (Figure 3C,D). Additionally, TRPV4 expression in the blood vessels was observed to be enhanced in the group treated with GSK1016790A (Figure 4).

### 3.3. Vascular Endothelial Growth Factor (VEGF) in OSCC Lesions

**Figure 5:**
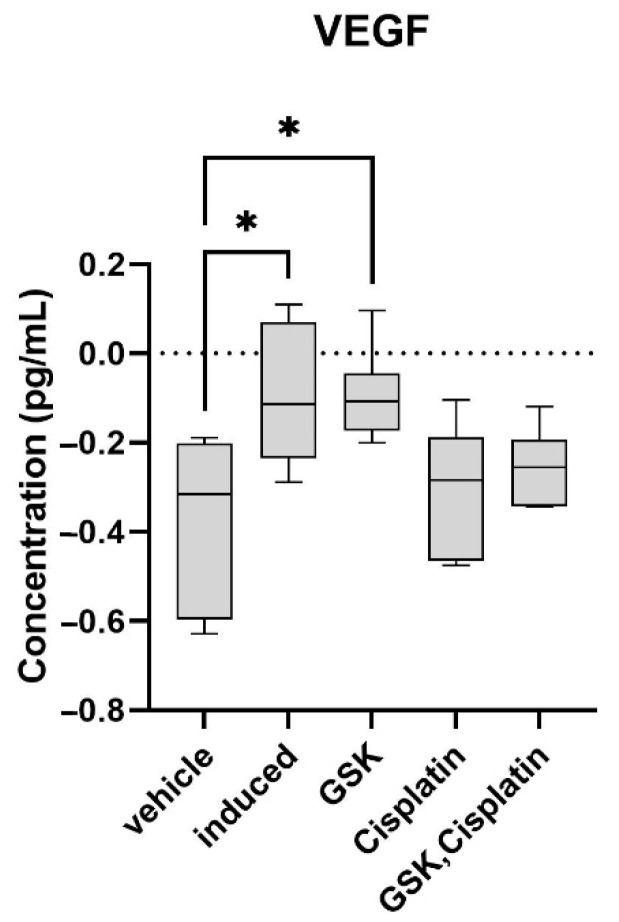
Vascular endothelial growth factor (VEGF) protein concentration (graph in log transform) of the animal groups in this study. The data are presented as mean ± CI. * *p* < 0.05.

Immunoassay results revealed that the VEGF protein concentration significantly increased (*p* < 0.05) in the induced-carcinoma group (0.850 ± 0.31 pg/mL) compared to the vehicle-treated group (0.455 ± 0.18 pg/mL) (Figure 5). Treatment with cisplatin (0.519 ± 0.17 pg/mL) or cisplatin + GSK1016790A (0.564 ± 0.11 pg/mL) decreased the VEGF protein concentration (Figure 5); however, the data were not statistically significant.

### 3.4. Angiopoietin/Tie Expression in OSCC Lesions

**Figure 6:**
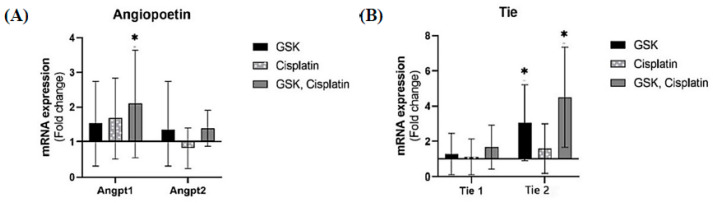
(**A**) Angiopoietins (Ang-1 and Ang-2) and (**B**) Tie (Tie-1 and Tie-2) expressions in tongue tissues using qPCR. Fold changes are relative to values obtained from the group induced with 4NQO only (set as 1) and normalized by changes in the average value of housekeeping genes (GAPDH, 18 s, and β-actin). Fold change values greater than 1 indicate an upregulation and vice versa. The data are presented as mean ± CI. * *p* < 0.05.

qPCR assay revealed that the mRNA level of Ang-1 increased in all treatment groups (Figure 6A) and is significantly increased (2.1-fold change, *p* < 0.05) in the cisplatin + GSK1016790A group. The Tie-2 mRNA level also significantly increased (4.5-fold change, *p* < 0.05) in the rat group treated with cisplatin + GSK1016790A (Figure 6B).

### 3.5. Microvessel Density and Microvessel-Pericyte-Coverage Index

**Figure 7:**
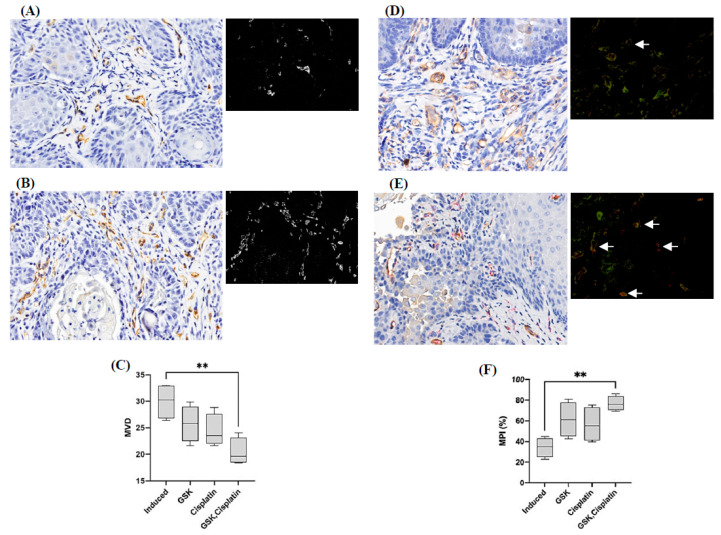
(**A**,**B**) Immunostaining of blood vessels of tongue tissues with OSCC using CD31. (**A**,**B**) are representative images of low and high microvessel density (MVD), respectively. Black and white images of A and B show vessels location. (**C**) Microvessel density in all treatment groups. (**D**,**E**) Double immunostaining of CD31 (blood vessels, brown) and α-sma (pericytes, red fuchsin) of tongue tissues with OSCC. (**D**,**E**) are representative images of low and high microvessel pericyte index (MPI), respectively. Black and white images of (**D**,**E**) show vessels location. White arrow shows pericytes and microvessels in close proximity to one another. (**F**) Microvessel pericyte index in all treatment groups. Scale bar is 50 µm. The data are presented as mean ± CI. ** *p* < 0.01.

Microvessel density significantly reduced (*p* < 0.01) in cisplatin + GSK1016790A-treated group (20.4 ± 2.597/high power field; 95% CI [16.27, 24.53]) compared to 4NQO-induced carcinoma group. Additionally, the microvessels-pericyte-coverage index significantly increased (*p* < 0.01) in cisplatin + GSK1016790A-treated group (76.83% ± 7.047; 95% CI [65.61, 88.04]) compared to the 4NQO-induced carcinoma group (Figure 7).

### 3.6. Tumor Size

**Figure 8:**
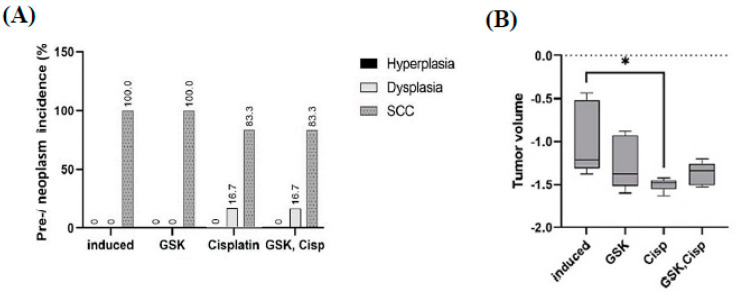
(**A**) Incidence of premalignant and malignant lesions in different groups. (**B**) Tumor volume (graph in log transform) in different groups. The data are presented as mean ± CI. * *p* < 0.05.

The 4NQO supplement produced premalignant and malignant lesions at the posterior region of the tongue. Administration of cisplatin as a single agent and in combination with GSK1016790A reduced the percentage of incidence of malignant lesions (Figure 8A). The tumor volume significantly reduced in the rat group treated with cisplatin alone (0.032 cm^3^, *p* < 0.05). Tumor volume was also greatly reduced in the cisplatin + GSK1016790A-treated group (0.042 cm^3^), although a statistically significant level was not achieved (Figure 8B).

## 4. Discussion

In this study, we combined the use of an anticancer drug, cisplatin, with a TRPV4 agonist and examined their effect on an animal model of oral squamous cell carcinoma. GSK1016790A was chosen as it is more potent compared to other commonly used TRPV4 agonists, such as 4αPDD [20]. The immunohistochemistry results obtained from this study revealed that TRPV4 expression was enhanced at the protein level in OSCC lesions compared to normal tissue. Our findings are in agreement with previous studies [21,22]. The TRPV4 mRNA level of expression was also upregulated in animals treated with GSK1016790A. The findings suggest that administration of GSK1016790A promotes TRPV4 expression, although the results obtained were not statistically significant.

Immunoassay results for VEGF revealed that the protein concentration of VEGF significantly increased in OSCC, and treatment with cisplatin or combination of cisplatin and a TRPV4 agonist reduced the VEGF level, although this reduction was not statistically significant.

In this study, we investigated the Angiopoietin/Tie expression in OSCC. Angiopoietin/Tie expression pathway is a vascular-specific receptor tyrosine kinase pathway and is considered an important pathway for vascular development, maturation, and endothelial cell survival [23,24,25,26,27]. Additionally, dysregulation in this pathway has been observed in many diseases, including cancer [28]. In this study, the administration of TRPV4 agonist, either as a single agent or in combination with cisplatin, resulted in a significant increase in Tie-2 mRNA level. Tie-2 expression in pericytes controls angiogenesis and vessel maturation, and that inactivation of Tie-2 in pericytes provokes the progression of tumor growth in a significant way [23]. We also demonstrated that the Ang-1 mRNA expression level significantly increased in the animals treated with TRPV4 agonist and cisplatin. These findings support the involvement of the Ang-1/Tie-2 system in angiogenesis of OSCC lesions when treated with TRPV4 agonist and cisplatin. In a related study, it was observed that Ang-1 promotes vessel stabilization without inducing vascular sprouting during neonatal development [24]. Similarly, in adult vasculature, the uncoupling of Ang-1 from its angiogenic growth factor such as vascular endothelial growth factor (VEGF), disrupted the integrity of the vessels [25]. A previous study of OSCC in humans reported that overexpression of Ang-1 enhances the interactions between the endothelial and periendothelial cells. It was suggested that overexpression of Ang-1 may be associated with more than 70% inhibition in the growth of OSCC [26].

On the contrary, Ang-2 is produced in inflammatory conditions and in endothelial cells of tumor vessels undergoing remodeling [27]. High Ang-2 expression has also been associated with destabilization of the vessels, resulting in vessel sprouting or vessel regression in the presence or absence of VEGF [29,30]. Previous studies have proven that blockade of Ang-2 results in a reduction in tumor vascularity, which is most likely brought about by suppressing vessel sprouting and/or lessening of the vessel size [31,32,33]. On the other hand, phosphorylation of Tie-2 is associated with the relative expression level of Ang-1 and Ang-2, wherein overexpression of Ang-1 will override the activity of Ang-2 from binding to Tie-2 [34]. While other pathways may be involved in promoting vessel maturation, the data obtained in this study with regards to the expression of Ang-1 and Tie-2 genes suggest that TRPV4 activation in OSCC may promote the maturation of tumor vessels via modulation of the Ang-1/Tie-2 pathway.

High microvessel density count in tumor lesions with high vascular density is mostly associated with poor prognosis or distant metastasis [35]. When TRPV4 agonist was combined with cisplatin, the significantly low microvessel density value observed in this study suggested that the activation of TRPV4 in combination with cisplatin was effective in reducing the formation of new blood vessels in OSCC. Thus, it is suggested that activation of TRPV4, along with cisplatin, may help with normal proliferation of endothelial cells.

The stabilization of the vessels in terms of pericyte coverage was also determined in this study, as vessel architecture is important, not only for the growth of tumor, but also for its crucial part in tumor metastasis [19,36,37]. The absence of pericytes or abnormal pericyte coverage in tumor microvessels has been hypothesized to increase the potential of metastasis through the presence of immature microvessels rather than mature vessels [19]. In this study, significant pericyte coverage was observed in the groups that received co-administration of TRPV4 agonist and cisplatin. The finding is supported by an earlier study, in which high pericytes-stabilized vessels were seen on tumor vessels following activation of TRPV4 [15]. It has been reported that patients who were categorized with a low microvessel pericyte index with high immature tumor microvessels were significantly associated with poor survival rate [19]. In addition, it was suggested that a high concentration of Ang-1 may be associated with recruiting pericytes to the immature vessels of the tumor region. In our findings, a higher level of Ang-1, rather than Ang-2, in the group treated with TRPV4 agonist and cisplatin indicates that Ang-1 may be involved in recruiting pericytes resulting in maturation of the blood vessels. As proposed in a previous study, inhibition of Ang-2 promotes the normalization of vessels, due, in part, to the unopposed action of Ang-1 [38].

Although, we examined the expression profile of Ang 1, 2 and Tie 1, 2 at the mRNA level, we did not examine their expression profile at the protein level. There is evidence, however, showing that Tie-2 expression in pericytes controls angiogenesis and vessel maturation [23]. We have examined the microvessel density and microvessel-pericyte-coverage index, and our findings reveal that microvessel density decreased significantly and the percentage of microvessels covered with pericytes increased significantly after treatment with GSK + cisplatin. These data relate to the results showing upregulation of TIE-2 mRNA expression in our study, implicating that Tie-2 activation (phosphorylation) may be more enhanced following treatment with GSK + cisplatin, leading to vessel maturation.

In the current study, tumor size was significantly reduced in the group treated with cisplatin alone. Tumor size was also greatly reduced in the group treated with the combination of a TRPV4 agonist and cisplatin; however, the reduction was not statistically significant. In this case, the sample size may not have been sufficient to obtain a statistically significant value, although the sample size was calculated using a formula [14].

Contemporarily, various treatment modalities are available for OSCC, which include resection/excision, radiotherapy, systemic cytotoxic chemotherapy, immunotherapy, and any integration of these, either concomitantly or sequentially [39,40,41]. Surgery, radiotherapy, and chemotherapy are the main treatment approaches for locally advanced cancer [39,40,41]. Among the various chemoradiotherapy regimens available, cisplatin-containing regimens are often regarded as the standard choice of treatment and remain some of the most widely used treatments [42]. Understanding the side effects of the different types of treatment regimens is equally important, as this may affect the preferred choice of treatment. In this study, we have not studied the side effects of the treatment regimens employed in this study; thus, further studies will be required. 

It has been reported that certain types of cancer behave and respond differently towards different types of treatment [39,40,41]. Due to the complex and unique anatomy of the head and neck region, limitations in surgical resection remain one of the main challenges, especially when the cancer lesions have progressed to an advanced stage. At this stage, even chemotherapy and radiotherapy treatment may not be able to offer the best desired outcome. Therefore, novel therapy strategies may offer an alternative treatment for head and neck cancer, especially when the cancer has advanced beyond the resectionable stage [43,44]. Recently, the use of targeted therapies for cancer treatment has been gaining interest [28,43,44]. Designing novel targeted therapies and understanding their molecular pathways of action can increase the ability to discover and design new targeted drugs. The findings of the effect of TRPV4 activation on OSCC or the effect of combination treatment of cisplatin and a TRPV4 agonist on OSCC observed in this study may help to develop new targeted therapies for OSCC.

## 5. Conclusions

While other pathways may be involved in promoting vessel maturation, the data obtained in this study suggest that a combination of a TRPV4 agonist with cisplatin for the treatment of OSCC promote vessels normalization via modulation of the Ang-1/Tie-2 pathway. Vascular normalization involves restoring deformities, both structurally and functionally, and this may lead to an improvement in the tumor microenvironment, as the responses towards various types of therapies, such as absorption of drugs, will be optimized.

## Figures and Tables

**Table 1:** Primer pairs of genes of interest used for RT^2^ Profiler arrays.

Symbols	Description	Refseq
Ang1	Angiopoietin 1	NM_053546
Ang2	Angiopoietin 2	NM_134454
Tie1	Tyrosine kinase with immunoglobulin-like and EGF-like domains 1	NM_053545
TRPV4	Transient receptor potential cation channel, subfamily vanilloid, member 4	NM_023970
Tek/Tie2	TEK tyrosine kinase, endothelial	NM_001105737
GAPDH	Glyceraldehyde-3-phosphate dehydrogenase	NM_017008
18SrRNA	Rat 18’s rRNA sequence	X01117
Actb	Beta-actin	NM_031144

**Table 2:** Optimization of primary antibodies used for immunohistochemistry staining.

Antibody	Dilution Ratio	Incubation Time (Minutes)	Company
TRPV4 (NB110-74960)	1:3500	30	Novus Biologicals, Littleton, CO, USA
CD 31 [EPR17259] (ab182981)	1:2000	30	Abcam, Cambridge, UK
Alpha smooth muscle actin (α-SMA) [EPR5368] (ab124964)	1:2000	30	Abcam, Cambridge, UK

## Data Availability

Data are available upon request.
